# FAO laboratory mapping tool results analysis for veterinary laboratories from 2012 to 2020: highlights of the gaps, the strengths across Southeast Asia and implications for capacity building activities

**DOI:** 10.3389/fvets.2025.1677993

**Published:** 2026-03-04

**Authors:** Morgane Gourlaouen, Stuart D. Blacksell, Khanh Kim Le, Christopher J. Morrissy, Wantanee Kalpravidh, Kachen Wongsathapornchai, Paolo Motta, Asfri Rangkuti, Gwenaelle Dauphin, Lidewij Wiersma, Thao Tran, Luca Porfiri, Beatrice Mouillé, Filip Claes

**Affiliations:** 1 Food and Agriculture Organization of the United Nations, Animal Health Services (NSAH) Laboratory Unit, Rome, Italy; 2 Mahidol-Oxford Tropical Medicine Research Unit, Faculty of Tropical Medicine, Mahidol University, Bangkok, Thailand; 3 Nuffield Department of Medicine, Centre for Tropical Medicine & Global Health, University of Oxford, Oxford, United Kingdom; 4 Food and Agriculture Organization of the United Nations, Regional Office for Asia and the Pacific, Emergency Center of the Transboundary Animal Diseases, Bangkok, Thailand

**Keywords:** LMT, capacity building, veterinary laboratory, ASEAN countries, evaluation

## Abstract

**Introduction:**

The Food and Agriculture Organization of the United Nations (FAO) developed the Laboratory Mapping Tool (FAO-LMT) Core module in 2010 to assess the general activities and capacities of central veterinary laboratories. Indeed, the FAO-LMT Core can determine strengths and gaps in laboratory functionality to define mechanisms and targets for capacity building to fill the measured gaps. This article analyzes FAO-LMT-Core evaluation data on 5 areas and 17 categories across 10 Southeast Asian countries, including 32 national and sub-national laboratories, from 2012 to 2020. This result creates a premise for improving, upgrading, and investing in laboratories. They also provide valuable and objective insight into the main constraints veterinary laboratories face in Southeast Asia.

**Objective:**

The article aims to identify and assess the gaps and strengths of veterinary laboratories in Southeast Asia.

**Methods:**

Thirty-two national and subnational laboratories participated in the assessment using the FAO’s Laboratory Mapping Tool (LMT)-Core module. The results of the LMT assessment were collected from 2012 to 2020, depending on each laboratory. All data from the 32 laboratories in the 10 countries have been reviewed, summarized, and analyzed from a regional perspective; for laboratories displaying several LMT-Core assessments, the results from the latest evaluation were used for this regional analysis.

**Results:**

The 32 laboratories from 10 ASEAN countries assessed in this study obtained an overall high result for the LMT-Core (mean average score = 60%; min: 23%, max: 82%). Most of them show a steady increase in the laboratory functionality of veterinary diagnostic laboratories. Laboratories already at a relatively higher level have managed to maintain their status, and those specifically targeted by support programs have increased their scores significantly.

**Conclusion:**

Efforts and investments in the laboratories included in this study have impacted the routine activity of these facilities, and the LMT-Core module can be applied to monitor the progress objectively and in a harmonized manner; the importance of using a standardized questionnaire is undeniable. To strengthen health security in the ASEAN region, it is crucial to maintain the current regional programs on quality assurance and biosafety/biosecurity, to encourage the various governments to ensure access to an adequate laboratory budget, to develop a veterinary laboratory policy and to link this policy to the ASEAN regional framework for laboratory capacity building and networking in ASEAN. It will help ensure the sustainable development of veterinary laboratories within ASEAN.

## Introduction

The continuing expansion of the human population, international trade, and ease of travel have dramatically increased the risks of transmission and exposure of humans and animals to a growing list of pathogens (1). Frequent outbreaks of emerging and re-emerging diseases in recent years raise concerns over the preparedness of animal and human health communities globally to respond safely and effectively to outbreaks of infectious diseases (2). Any disease surveillance or control program relies on solid laboratory capacities and capabilities (3, 4). Veterinary laboratories (VLs) that may be local, national, or regional are tasked with early detection and diagnosis of endemic disease agents and new emerging pathogens, thus playing a critical role in protecting both animal and human health and, subsequently, in the economic stability related to animal production (5, 6). Although VLs inevitably handle potential pathogens on a routine basis, their role is often underestimated and disregarded, and VLs truly lack attention and funding. This is particularly true in low and middle-income countries, where animal diseases are highly damageable and impactful due to a vicious circle of neglect (7–9). Within its mandate, the Food and Agriculture Organization of the United Nations (FAO of the UN) is dedicated to strengthening the capacities of VLs in Member States countries to tackle the vicious circle of neglect by providing the support required by VLs for their adequate and safe functioning (10). Because the settings of VLs can dramatically vary from one facility to another, the starting point for appropriate capacity-building activities is needs assessment to specifically map out the gaps and the strengths of each laboratory. Then, a stepwise approach is followed: based on the assessed needs, a tailored capacity-building program can be designed and implemented. Reporting of the efforts and improvement of the situation can be used as advocacy material to consolidate awareness and fundraising for VLs. A needs assessment can then be performed again to re-evaluate the situation and measure the progression over time (11–13).

The FAO Laboratory Mapping Tool (LMT) was developed in 2010 under the Emerging Pandemic Threats Program (2009–2014) (14). The FAO LMT-Core module aims to assess the generic laboratory capacities, capabilities and functionality. It is based on a standardized questionnaire that allows data to be captured by external evaluators or self-assessments. The tool is designed to facilitate the assessment of veterinary laboratories in a systematic and semi-quantitative manner, and results are presented in both tabular and graphical formats. The tool can be used by any veterinary laboratory in any region or by any development partner working on veterinary laboratory capacity building to assess a given laboratory functionality and identify priorities and gaps standardized. It also allows the development of a standard baseline for laboratories within a defined region, which will help build a regional approach. The outcome of the LMT reflects a “snapshot” view of a given laboratory’s level of functionality and can measure its evolution over time. It shows a summary profile or “map” and spider graphs, allowing users to easily visualize and compare the laboratory results to previous assessments or other laboratories. Several articles reporting the results obtained with the FAO LMT-Core in capacity building have been published over the years (15, 16). Hereby, we present the results of 8 years of assessments and the progression measurement of laboratory capacities and capabilities in 32 laboratories in the ASEAN region. To our knowledge, this is the first time that an analysis of veterinary laboratory capacities in the region has been evaluated. Many of the positive outcomes are also discussed hereby. This article not only aims to present how the LMT-Core can be applied at national and regional levels, but the authors also wish to demonstrate and discuss how the data can be extracted and used for efforts and financial advocacy.

## Methods

### Scope of the study

**Figure 1:**
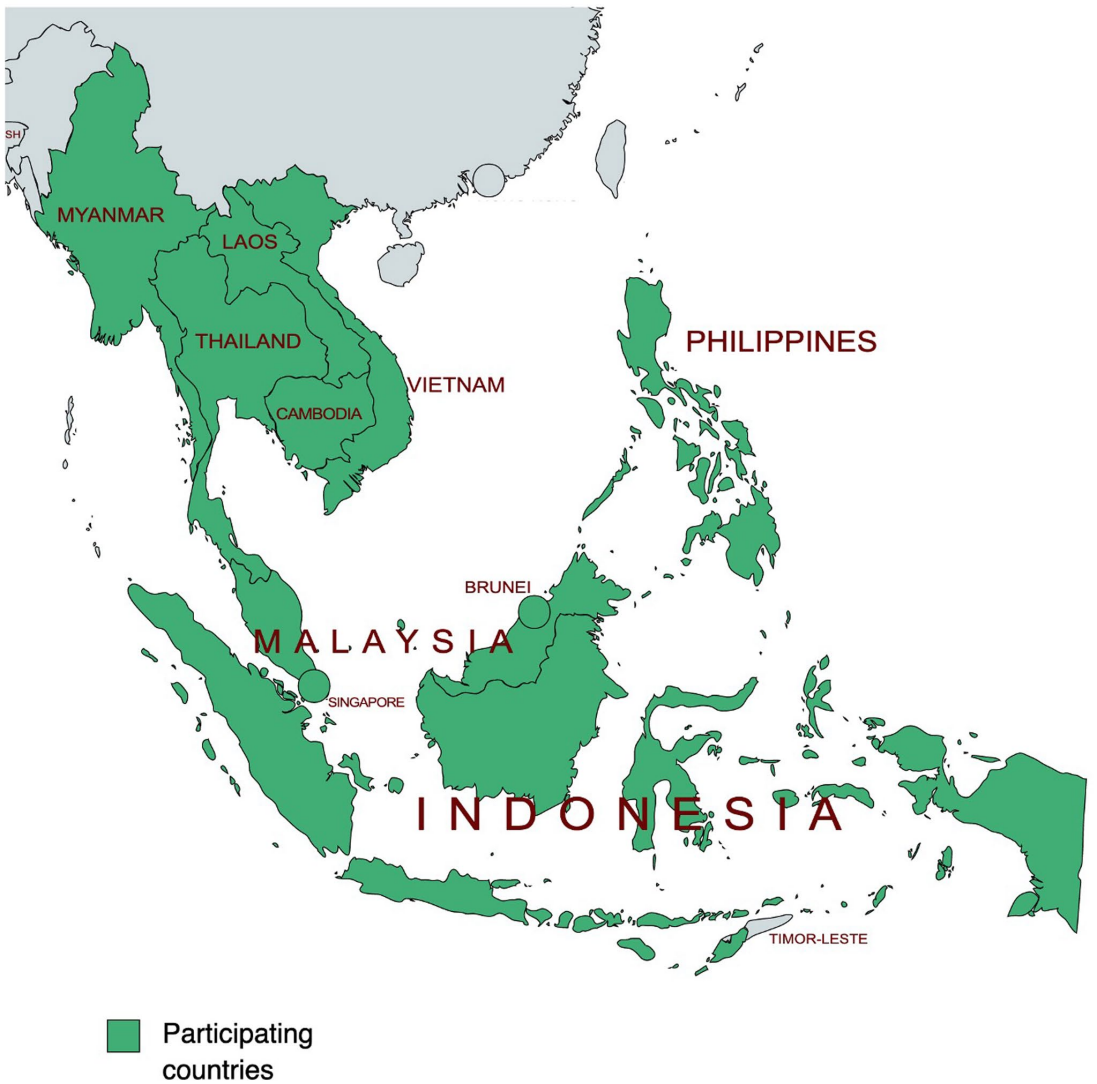
Map of the participating countries in the ASEAN region. In the map, green indicates the countries participating in this study where laboratories have been assessed using the LMT-Core. The map was made on a website: www.mapchart.net.

Thirty-two (32) VLs across ten (10) countries in the ASEAN region (Figure 1), including local, sub-national and national laboratories, were assessed using the FAO LMT-Core. The VLs participating in the assessment are presented in the table in Figure 1. The LMT assessments and data collection were performed from 2012 until 2020 by external laboratory experts using the latest available version of the LMT Excel spreadsheet.

### Harmonized assessment using the LMT-Core

**Figure 2:**
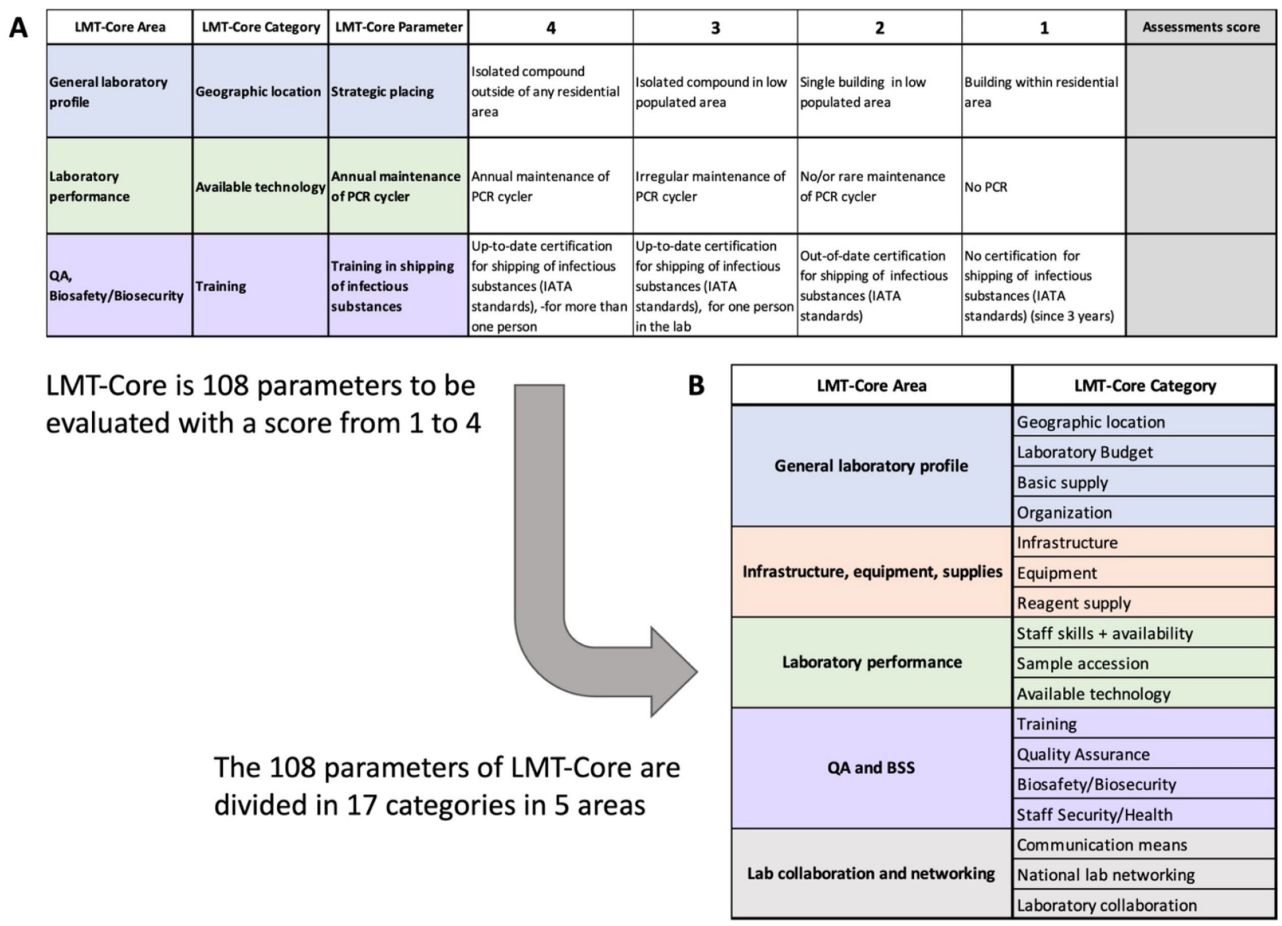
The Standardized LMT-Core questionnaire. Top panel **A**: Example of three (3) LMT-Core parameters related to the strategic placing, the annual maintenance of the PCR cycler and the training in shipping infectious substances. For each parameter, 4 scenarios are described with an associated score from 1 (worst) to 4 (best). Based on their observations, the LMT-Core assessors must choose the scenario most suited to the laboratory under assessment and attribute the associated scoring for the evaluated parameter. Bottom panel **B**: The LMT-Core 108 parameters are divided into 17 LMT-Core categories and 5 LMT-Core Areas. Upon completion of the LMT-Core questionnaire by the assessors, cumulative scores are calculated for each category and each area.

**Figure 3:**
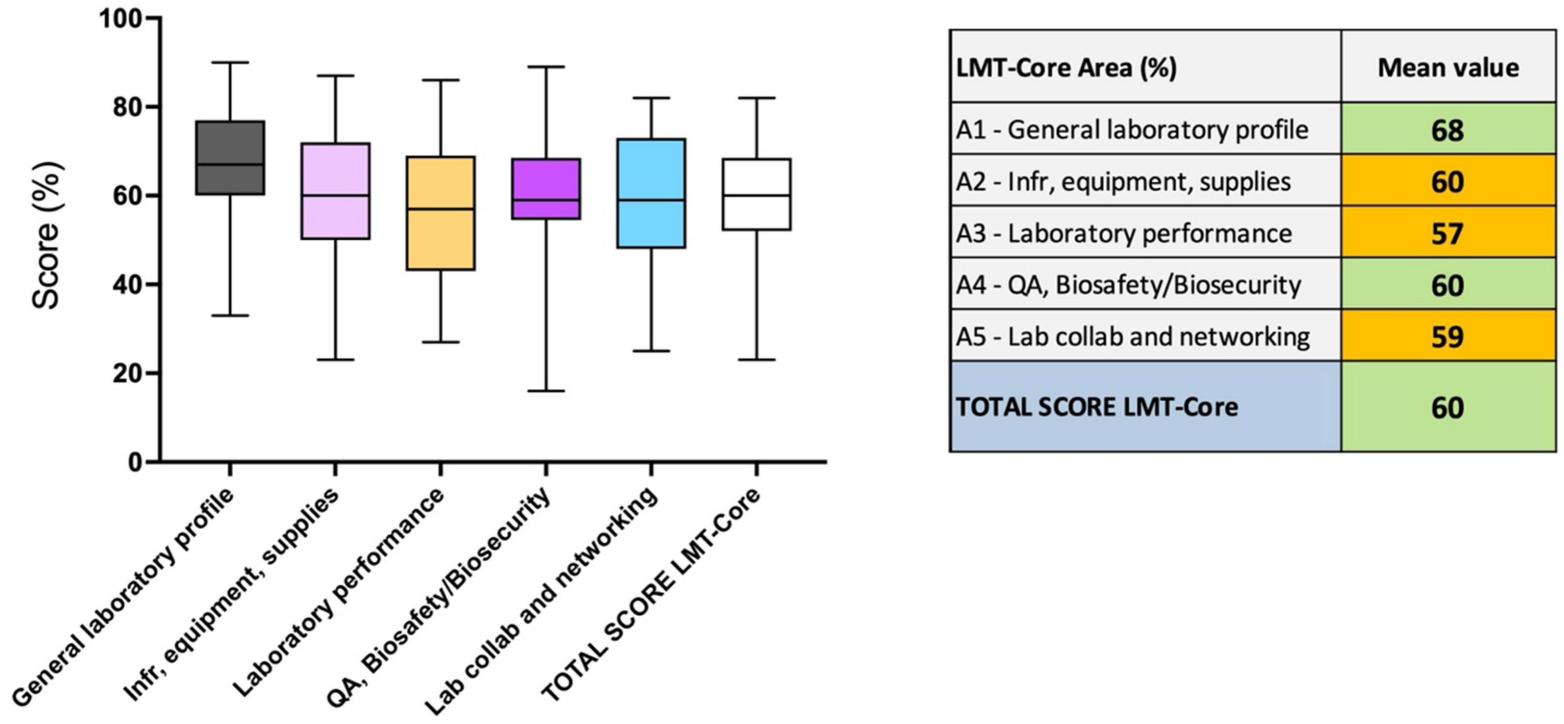
Regional results analysis of 32 laboratories in the ASEAN region. The table on the right presents the results (%) of the overall LMT-Core score and mean values*. The box plot graph was edited to visualize the data collected for the LMT-Core areas and overall scores in the table above. The box extends from the 25th to 75th percentiles, and the whiskers are plotted at the minimum and maximum values. The line in the middle of the box is plotted at the median. * For laboratories with several assessments, the latest results were used for the mean value calculation.

LMT-Core is developed in a semi-automated Excel spreadsheet where 108 parameters related to veterinary laboratory capacities and capabilities can be evaluated. Similar to a quiz, each parameter suggests 4 scenarios with associated scoring from 1 to 4, where 1 is considered the worst scenario, and 4 is the best “ideal” scenario (Figure 2A, upper panel). These hundred and eight (108) parameters are distributed into seventeen (17) categories, which are composed of the five LMT-Core areas, i.e., general profile; infrastructure, equipment, and supplies; laboratory performance; quality assurance, biosafety, and biosecurity (QA and BSS); and laboratory collaboration and networking (Figure 2B, lower panel). The assessor filling up the LMT-Core selects the most appropriate scenario; in other words, the assessor provides a score of 1 to 4 to each parameter, and once all parameters have been filled, scoring is automatically calculated for each category, each area and the overall LMT-Core; the scores are presented in percentages (Figure 3).

### Data collection and analyses

Thirty-two VLs were visited in ASEAN, and LMT assessments were applied to all of them. Hereby, the analysis of the results was performed as a regional analysis, where the latest evaluation of the participating laboratories is compared and analysed for common gaps and strengths. Statistical Assessment of Data Normality: To evaluate whether the results from the 32 participating laboratories across the 17 sections of the LMT-Core module followed a normal distribution, we applied the Shapiro–Wilk test, a robust method for assessing normality in small-to-moderate sample sizes (*n* < 50). This non-parametric test was selected due to its high sensitivity to deviations from normality, particularly in datasets with limited observations per group. For each parameter, the null hypothesis (H₀: data are normally distributed) was tested at a significance threshold of *α* = 0.05. Whilst the results of thirteen LMT categories passed this normal test, the results of four parameters, i.e., Laboratory budget, basic supply, organization and biosafety/biosecurity did not pass the test (Supplementary Table 1). These results can be easily explained when considering the limited variability in the results for the categories of *Laboratory Budget*, *Basic Supplies*, and *Organization*. In these areas, the assessed laboratories frequently exhibited similar performance. For instance, the vast majority of laboratories had established organizational charts with clear staff role descriptions, leading to consistent scores across these specific indicators. Finally, the overall LMT-Core results of each laboratory were also tested using the Shapiro–Wilk test and the results followed a normal distribution (W = 0.9646; *p* value = 0.3647).

The mean values were calculated for each country and region for each category, area and overall LMT-Core. Graphs were plotted either in Excel or PRISM for the box plot.

For the eighteen (18) laboratories that were assessed more than once, a Δ score was calculated by simply subtracting the scores obtained at the most recent and obtained at the first assessments.

To enable a more straightforward interpretation of the LMT-Core results analysis, a scoring scale with associated color code is applied as follows:

0–20.0% = very weak (dark red);
20.1–40.0% = weak (red);
40.1–60.0% = average (orange);
60.1–80.0% = strong (light green);
80.1–100% = very strong (dark green).

## Results

### Regional overview of LMT-Core results and analysis of the results collected between 2012 and 2020

The mean value for the regional analysis of the LMT-Core assessments across the 32 laboratories included in this study is 60%. The box plot indicates that most results fall between 55 and 65%, with the lowest mean result at 23% and the highest at 82% (Figure 3 and Supplementary Table 2). In the lower range of the chart (below 40%), one outlier laboratory with the mean value of 23%, while in the upper range (above 60%), 16 laboratories are represented. The latest assessment results were used for laboratories that were assessed several times, and for 21 out of the 32 laboratories, the assessments were performed in 2019 or 2020. The calculated mean values demonstrate a medium-high range of results for the LMT-Core areas. The *general laboratory profile* and *QA and BSS* scored 68% (range 33 to 90%) and 60%, respectively, and are highlighted in light green. The *infrastructure, Equipment and Supplies*, *Laboratory performance* and *Laboratory collaboration and networking* scored 60% (range 23 to 87%), 57% (range 27 to 86%) and 59% (range 25 to 82%), respectively; these mean values are highlighted in orange. The box plot graph shows a homogeneity of the median values of each LMT-Core area and overall LMT-Core score. The *QA, BS/BS* area has the longest whiskers; the lowest value for this category is 16% and the highest is 89% (Figure 3 and Supplementary Table 2).

### LMT-Core analysis—area per area

#### Area 1 (A1)—general laboratory profile

**Figure 4:**
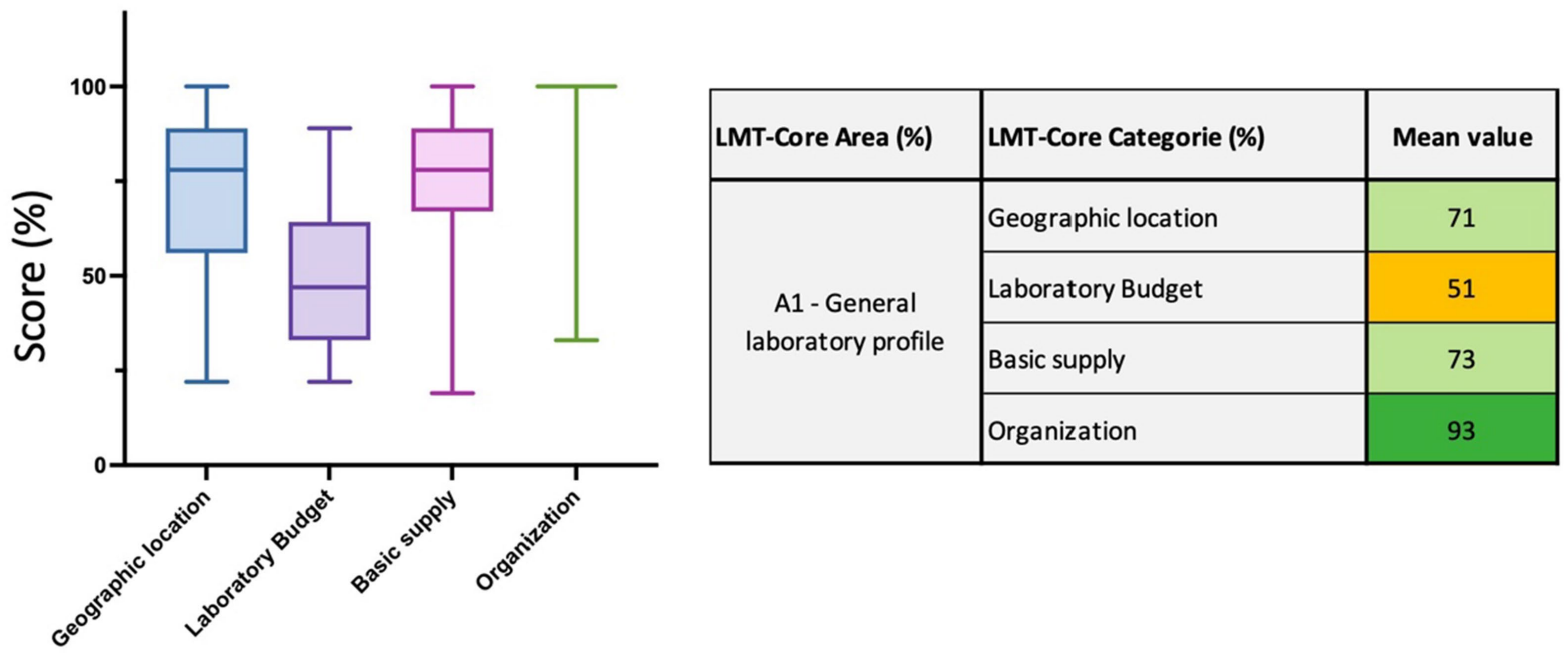
LMT-Core results analysis for Area 1, general laboratory profile. The table on the right presents the mean value results (%) of the LMT-Core categories within A1. The box plot graph was edited to visualize all the data collected for the 4 LMT-Core categories in A1. The box extends from the 25th to 75th percentiles, and the whiskers are plotted at the minimum and maximum values. The line in the middle of the box is plotted at the median.

Within this first area of the LMT-Core, 10 parameters spread out in four categories are assessed. The first category, namely the *geographic location,* assessed whether the laboratories have an appropriate location (far from residential areas) and are easily accessible. The second category aims to evaluate the *laboratory budget* to understand whether laboratories have any financial autonomy for diagnostic routine activity, research activity, and infrastructure maintenance. The third category, namely *basic supply,* evaluates the access to electricity and water. Finally, the fourth category, *organization,* looks at the staff organization system and clearly describes their respective responsibilities. Figure 4 shows that the category *laboratory budget* scored the weakest results here, with 51% (range 22 to 89%). The box plot clearly shows that most of the laboratories scored lower in this category compared to the other three categories. The *geographic location* (71% range 22 to 100%), *basic supply* (73% range 19 to 100%), and *organization* (93% range 33 to 100%) categories demonstrate here strong capacities at the regional level. There is no visible plot box for *organization* since 26 out of the 32 laboratories scored 100% for this category (Figure 4 and Supplementary Table 2).

#### Area 2 (A2)—infrastructure, equipment and reagent supply

**Figure 5:**
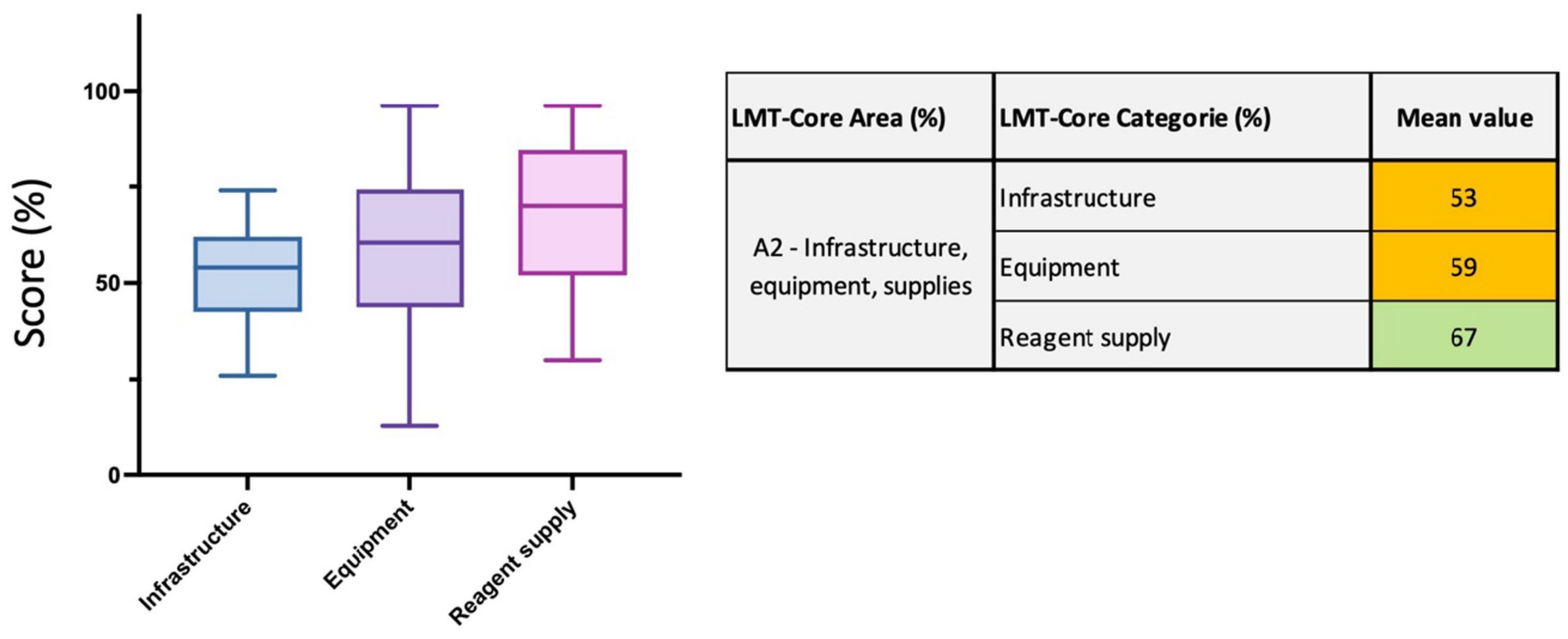
LMT-Core results analysis for Area 2, infrastructure, equipment, supplies. The table on the right presents the mean value results (%) of the LMT-Core categories within A2. The box plot graph was edited to visualize all the data collected for the 3 LMT-Core categories in A2. The box extends from the 25th to 75th percentiles, and the whiskers are plotted at the minimum and maximum values. The line in the middle of the box is plotted at the median.

In this area, 26 parameters are divided into 3 categories to assess the *infrastructure, equipment and reagent supply capacities.* The parameters within *infrastructure* evaluates the quality of the laboratory rooms (general state, allocated space and maintenance) with the availability of (i) locker room(s) for the staff to change and store valuables, (ii) sterilizing and storage rooms, (iii) a cleaning plan in place, (iv) air conditioning and possibly an airlock system, (v) adapted biosafety levels in the different laboratory departments, (vi) clearly separated laboratory departments and controlled access, (vii) emergency exit, alarm system and adapted equipment to respond to fire hazard. The parameters within the *equipment* category evaluate the quantity and the quality of the laboratory equipment in the different laboratory departments to ensure that sample testing can be performed in the most efficient and safest way. The parameters in the *reagent supply* evaluate the availability of reagents and consumables and whether the laboratory is autonomous for reagent procurement. Importantly, the storage, validity and traceability of reagents with respect to the ISO17025 standard are also assessed. In this regional analysis, the *infrastructure* category scored 53%, the *equipment* scored 59%, and the *reagent supply* scored 67% (range 30 to 96%). The *infrastructure* category presents limited whiskers, the lowest result being 26% and the highest 74%. The range variation is higher for the *equipment* category; the lowest result is 13% and the highest is 96% (Figure 5 and Supplementary Table 2).

#### Area 3 (A3)—laboratory performance

**Figure 6:**
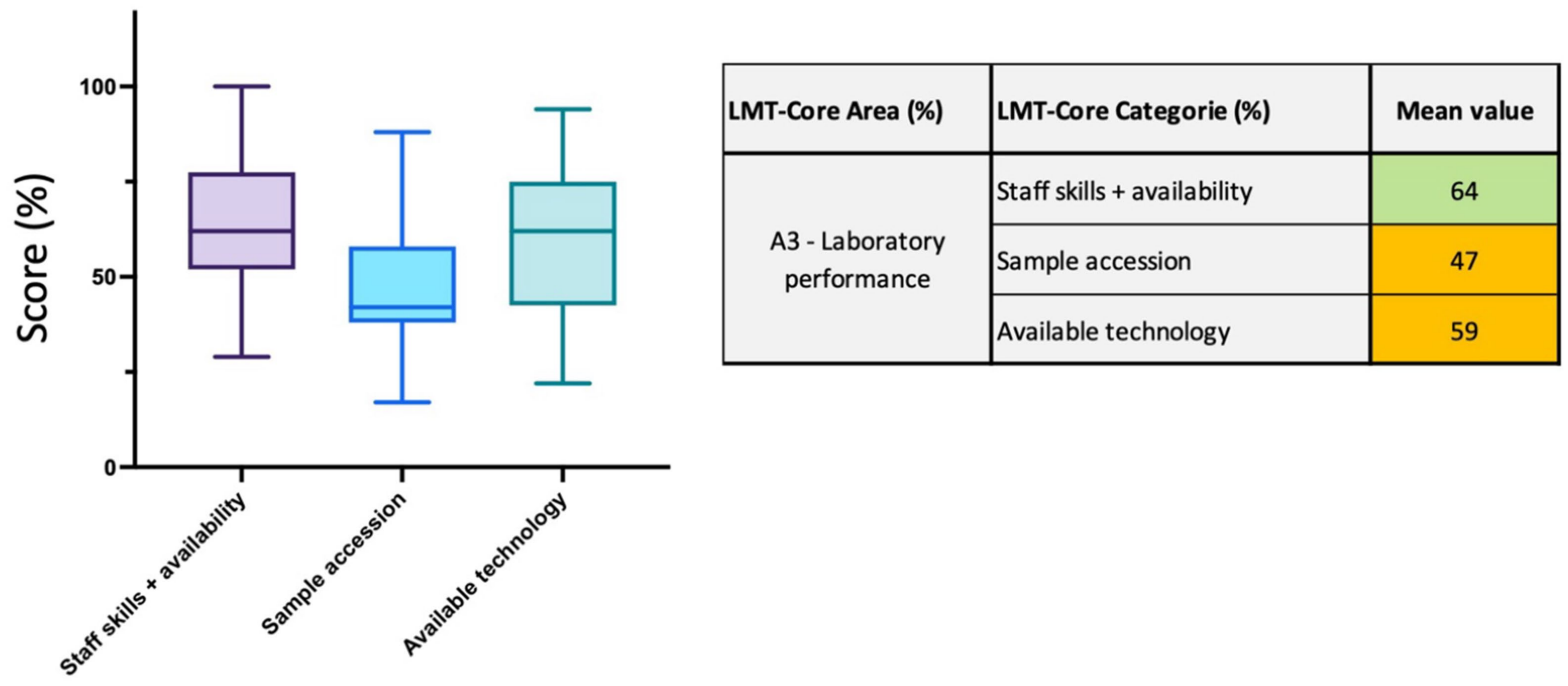
LMT-Core results analysis for Area 3, laboratory performance. The table on the right presents the mean value results (%) of the LMT-Core categories within A3. The box plot graph was edited to visualize all the data collected for the 3 LMT-Core categories in A3. The box extends from the 25th to 75th percentiles, and the whiskers are plotted at the minimum and maximum values. The line in the middle of the box is plotted at the median.

In this area, 27 parameters are divided into 3 categories: *staff skills and availability*, *sample accession*, and *available technology* to assess the capacities related to the *laboratory performance.* In the *staff skills and availability,* the parameters determine the level of expertise of the laboratory staff for the different departments and testing procedures applied; for example, the expertise in post-mortem and necropsy procedures is very different from the expertise in molecular biology for PCR testing. The availability of the staff in case of outbreaks and emergencies is also evaluated. The regional analysis shows that the veterinary laboratories in ASEAN have access to appropriate staff skills with a mean value of 64% ranging from 29 to 100% (Figure 6 and Supplementary Table 2). The parameters in *sample accession* assess the number of samples received by the different departments and maintenance of testing skills, and here, the mean value is 47% ranging from 17 to 88%. Thirteen laboratories scored low results in this category (<40%) (Figure 6 and Supplementary Table 2). This category scored the lowest mean result in this regional study. Finally, the parameters in *available technology* evaluate the level of capacities and capabilities in place to perform a variety of tests for the different departments, such as Enzyme-Linked Immuno-Sorbent Assay (ELISA), Hemagglutination Inhibition (HI) test, immuno-fluorescence staining, PCR and sequencing. The mean value for *available technology* is 59% ranging from 22 to 94% (Figure 6 and Supplementary Table 2).

#### Area 4 (A4)—quality assurance (QA), biosafety and biosecurity (BSS)

**Figure 7:**
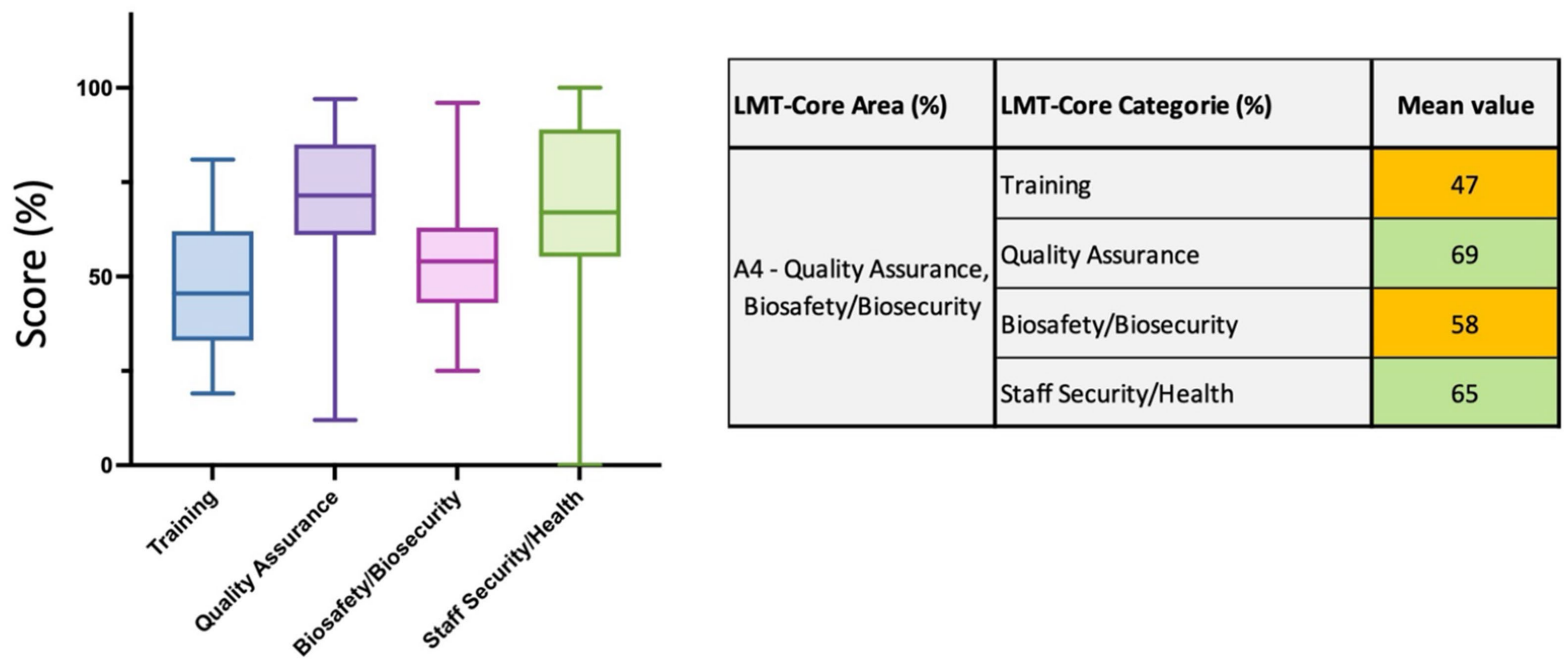
LMT-Core results analysis for Area 4, quality assurance and biosafety biosecurity. The table on the right presents the mean value results (%) of the LMT-Core categories within A4. The box plot graph was edited to visualize all the data collected for the 4 LMT-Core categories in A4. The box extends from the 25th to 75th percentiles, and the whiskers are plotted at the minimum and maximum values. The line in the middle of the box is plotted at the median.

In this critical area of the LMT-Core focusing on QA and BSS, 29 parameters are assessed and divided into 4 categories: *Training, QA, BSS, Staff security and Health*. The *training* category evaluates the training opportunities, internal and external, for Good Laboratory Practices (GLPs), diagnostic procedures, QA, BSS, maintenance and calibration, lab management and shipping of infectious substances (17). This category also scored the lowest results of this LMT-Core regional analysis with 47% ranging from 19 to 81% (Figure 7 and Supplementary Table 2). For the category *QA,* the mean value is 69% ranging from 12 to 97% and with only 2 laboratories with scores highlighted in red (<40%) (Figure 7 and Supplementary Table 2), thus showing that the laboratories have a good understanding and application of the ISO17025 standard. The category *BSS* scored 58% ranging from 25 to 96% (Figure 7 and Supplementary Table 2), and the parameters in this category assess the management of BSS capacities with the appointment of a BSS officer and the use of risk assessment to define clearly written procedures and ensure appropriate staff behaviors. The availability of Personal Protective Equipment (PPE), the maintenance of the BSCs, the measures against unintentional release of pathogens and the waste management in place are also evaluated in this category. Finally, the *staff security and health* category analyzes the appropriate access to occupational health, including pre-exposure and post-exposure vaccination; this category scored 65% ranging from 0 to 100% (Figure 7 and Supplementary Table 2).

#### 3.2.5 Area 5 (A5)—laboratory collaboration and networking

**Figure 8:**
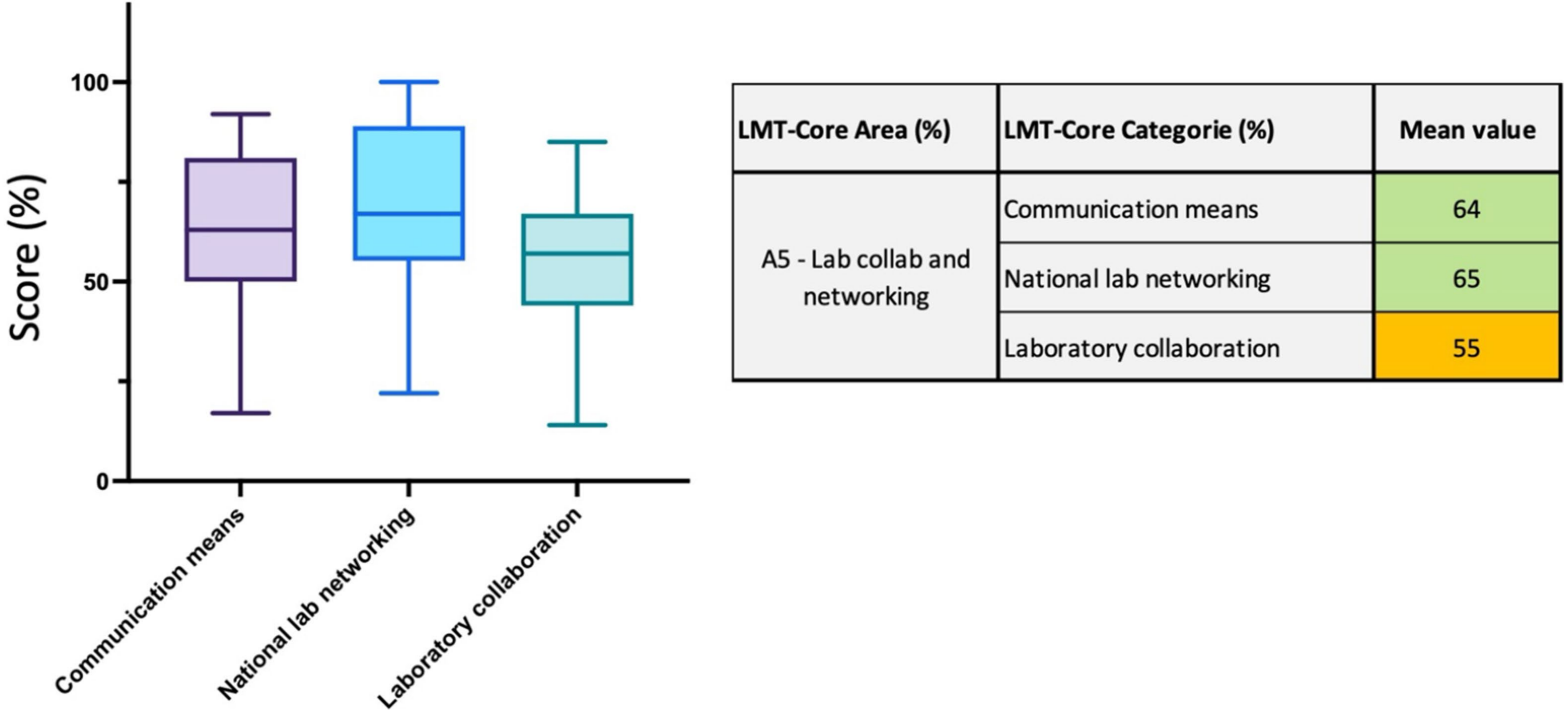
LMT-Core results analysis for Area 5, Laboratory collaboration and network. The table on the right presents the mean value results (%) of the LMT-Core categories within A5. The box plot graph was edited to visualize all the data collected for the 3 LMT-Core categories in A5. The box extends from the 25th to 75th percentiles, and the whiskers are plotted at the minimum and maximum values. The line in the middle of the box is plotted at the median.

In this area, 16 parameters are divided into 3 categories: *communication means*, *national lab networkin*g, and *laboratory collaboration*. While the first two categories scored 64% (ranging from 17 to 92%) and 65% (range 22 to 100%), indicating strong capacities in terms of access to phones, Wi-Fi, scientific publications, and communication within the national laboratory network, the parameters related to laboratory collaboration scored slightly lower at 55% (range 14 to 85%) (Figure 8 and Supplementary Table 2). The parameters within this category assess the communication with the laboratory network at regional and international levels and the level of information and data exchange with the national epidemiological unit (if existing).

### LMT assessments over the years—delta score analysis

**Figure 9:**
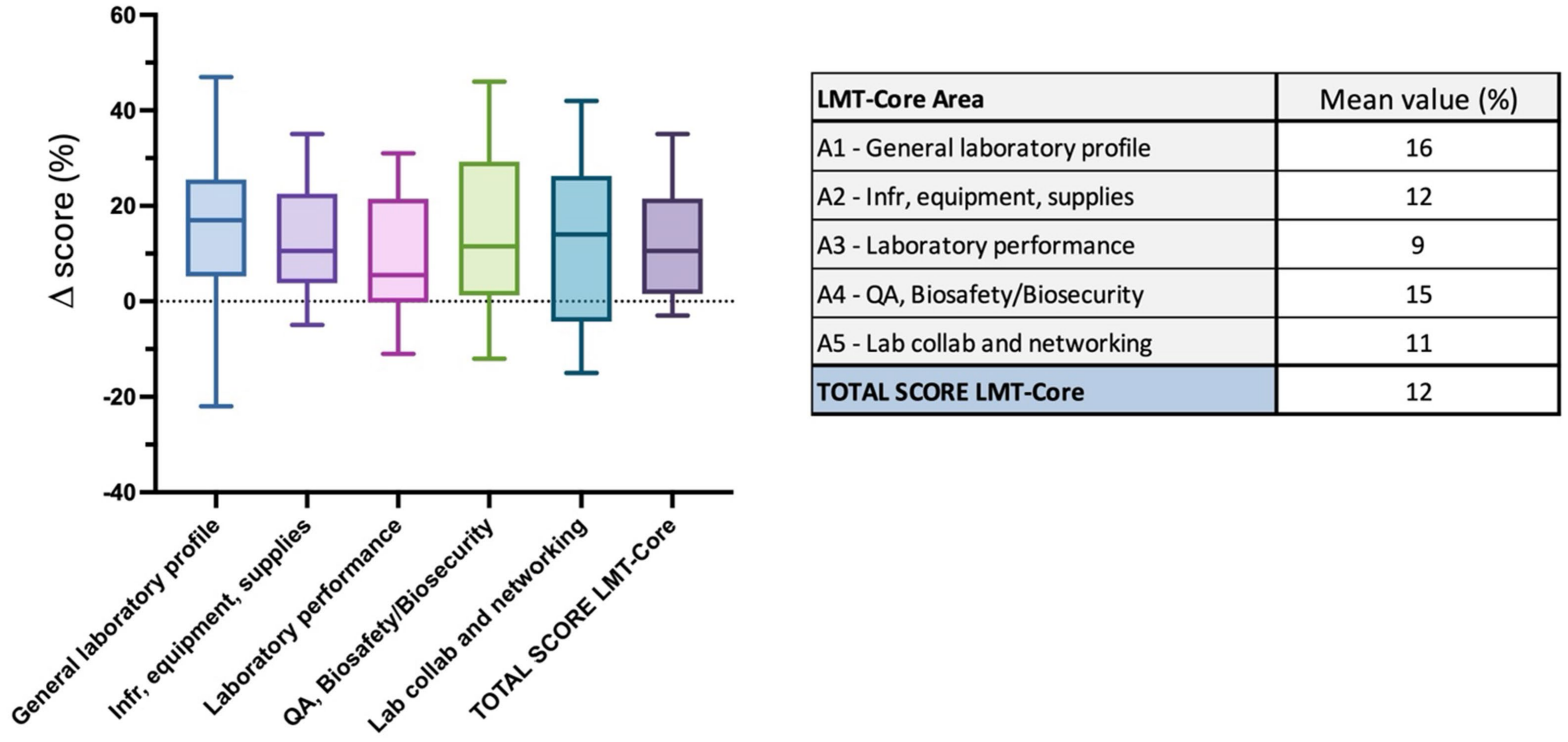
LMT-Core (areas) *Δ* results analysis of 18 laboratories. The table on the right presents the mean value Δ score (%) of the LMT-Core areas and overall results. The box plot graph was edited to visualize all the data collected. The box extends from the 25th to 75th percentiles, and the whiskers are plotted at the minimum and maximum values. The line in the middle of the box is plotted at the median.

In this study, 18 laboratories in 8 countries were assessed at least twice, thus providing monitoring of the LMT results over time. Some laboratories were assessed more than twice; therefore, for the Δ score results, the first and last assessments were used. For all laboratories, the assessments were performed several years apart thus giving time to properly measure any trend progress or decrease. For most laboratories, the first assessment occurred in 2012, while the second was in 2019. The overall LMT-Core results show an average increase of 12% (range −3 to 35%) between the first and last assessment (Figure 9 and Supplementary Table 3). The trend was positive for most laboratories, i.e., 2 laboratories had a negative trend, and 2 laboratories had a null Δ score. This demonstrates a general improvement in the capacities and capabilities of veterinary laboratories. More specifically, the areas *A1- General laboratory profile* and *A4- QA, BSS* improved by 16% (ranging from −22 to 47%) and 15% (ranging from −12 to 46%), respectively (Figure 9 and Supplementary Table 3). Here again, discrepancies between laboratories were observed, with some laboratories displaying a strong positive trend with Δ score results over 30 and 40%, while others had a negative trend over time (Figure 9 and Supplementary Table 3). The areas *A2- infrastructure, equipment and supplies*, *A3- Laboratory performance* and *A5- Lab collaboration and networking* also had positive Δ scores of 12% (ranging from −5% and to 35%), 9% (ranging from −11 to 30%) and 11% (ranging from −15 to 42%), respectively (Figure 9 and Supplementary Table 3).

**Figure 10:**
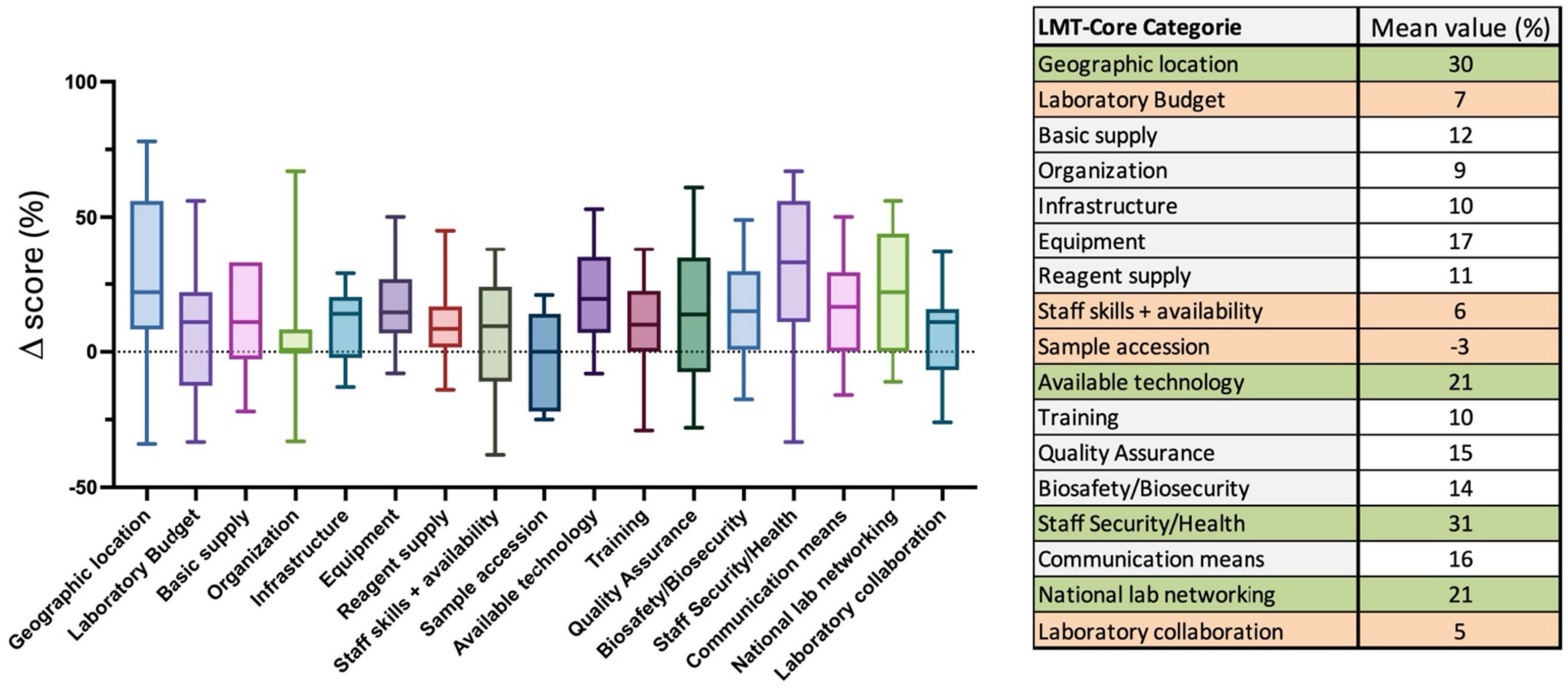
LMT-Core (categories) Δ results analysis of 18 laboratories. The table on the right presents the mean value Δ score (%) of the LMT-Core categories. The categories in green have a mean value Δ score > of 20%, while the ones in orange have a mean value Δ score < of 10%. The box plot graph was edited to visualize all the data collected. The actual box extends from the 25th to 75th percentiles, and the whiskers are plotted to the minimum and up to the maximum value. The line in the middle of the box is plotted at the median.

Focusing on the LMT categories, the four categories with the highest increase in their Δ score, shown in green in the table below (Figure 10), are *Geographic location* (30% range −34 to 78%), *Available technology* (21% range −8 to 53%), *Staff Security/health* (31% range −33 to 67%) and *National lab networking* (21% range −11 to 56%) (Figure 10 and Supplementary Table 3). The four categories that had the weakest increase, shown in orange in the table below (Figure 10), are *Laboratory budget* (7% range −33 to 56%), *Staff skills and availability* (6% range −38 to 38%), *Sample accession* (3% range −25 to 21%) and *Laboratory collaboration* (5% range −26 to 37%) (Figure 10 and Supplementary Table 3). The mean values Δ score results of the other categories spread out between 9 and 17% (Figure 10).

## Discussion

The FAO LMT is a family of standardized tools with several modules, e.g., LMT-Core, LMT-safety, LMT-AMR and LMT-Bacteriology (part of FAO-ATLASS), LMT-Biothreat and some disease-specific LMT modules are under development. The FAO LMT-Core module is used to measure the general capacities and overall functionalities of veterinary laboratories, and hereby, the results collected from the assessments of 32 laboratories that took place between 2012 and 2020 across ten Member States of ASEAN are presented and analyzed. LMT-Core results provide an overview of the general status of (i) individual veterinary laboratories, (ii) any laboratory network at the national level, and, of course, the results can also be extrapolated to a region. The evolution of the laboratories’ functionality can be followed over time, and applying the LMT-Core every year or every other year is generally recommended. This statement is verified by the detailed monitoring achieved in this study for the laboratories with several assessments. Ultimately, one of our observations is that the LMT-Core results are used to assist decision-makers in better defining the targets and mechanisms for both local and global laboratory capability and capacity-building. The LMT-Core was originally developed in 2010 to support laboratory capacity-building projects and following its successful implementation in veterinary laboratories across the globe, it became highly appreciated due to its simplicity and usefulness in mapping gaps and strengths. Another positive outcome is the expansion of a community of LMT assessors globally made of specifically trained laboratory experts and laboratory staff from Member States, which has flourished over the years. In this study, the assessments were performed by four assessors under the umbrella of FAO laboratory specialist activities. Whilst small results variation between assessors is undeniable due to differences caused by human perception, attitude or behaviors, e.g., some assessors can be more or less strict, thus the thorough questionnaire with detailed scenario-based answers should limit any judgmental opinion in the assessments and the results provided. The assessors also collaborated closely to ensure better harmonization, both during training workshops for Member States and while conducting joint assessments (18). They continuously discussed specific scenarios to reach consensus on scoring whenever doubts arose. Since the assessors performed most of the LMT-Core missions and evaluations together—and given that the LMT-Core comprises 108 parameters—any variation in scoring could be considered minimal. For instance, a variation in the scoring of 4 or 5 parameters out of the 108 evaluated would have little impact on the final LMT-Core score. Finally, the LMT evaluation process typically begins with an internal self-assessment conducted by laboratory staff. This self-assessment is then compared with the external scores provided by the assessors. This approach ensures that the scenario selected for each parameter is the one that best reflects the actual situation, regardless of whether it is chosen by the lab staff or the external LMT assessor. Those discussion and comparison between auto and external evaluation are always reported as useful and rich by both parties since it helps choosing the most suited scenarios for the laboratory under evaluation, it can also highlight hidden gaps or strengths but more importantly, it is the trigger for action plan discussion to fill the gaps.

The overall LMT-Core results of the 32 laboratories showed a mean value of 60% ranging from 23 to 82%. The Supplementary Table 2 shows the presence of one outlier, i.e., the laboratory 26 that scored 23% which is the only laboratory with a score in the low range (<40%—red). Fifteen laboratories scored an average score (40–60%—orange) and sixteen laboratories scored high results (>60%—green). The laboratories that participated in this study did not have equal access to public funding or government support. The ten countries involved can be grouped into high-, upper-middle, and lower-middle income categories. While we examined whether a correlation existed between national income and LMT-Core scores, variations in results could not be explained solely by GDP per capita (data not shown for data privacy reason). Notably, laboratories from higher-income countries did not consistently outperform those from lower-income countries. Several of the highest-scoring laboratories were located in lower-middle income settings, whereas the weakest performer—Lab 26, with a score of 23%—was not from a lower-middle income country. Although laboratories in high-income countries generally operate in more favorable environments with greater access to resources, our findings indicate that income level did not determine LMT-Core performance. The Δ score analysis also confirms this observation since the *laboratory budget* category was one of the categories that progressed the least whilst many other categories still considerably improved. It is worth noting that the distribution of laboratories by GDP per capita is uneven: only are from 3 laboratories are from high-income countries, the rest are split between lower middle-income and upper middle-income countries. Our observation is in agreement with an important report on *Biological Security Priorities in Southeast Asia* where the results from the WHO Joint External Evaluations (JEEs) and the WOAH Performance of Veterinary Service (PVS) Evaluation showed that variations between countries could only be partly explained by the socio-economic context (19). Whilst high-income countries, i.e., Singapore or Brunei, generally benefit from greater public resources, this advantage does not automatically translate into better evaluation results, as other factors come into play such as the political commitment and prioritization of biological security, the regional cooperation, the capacity to implement regulations or also the presence of collaboration or projects with international organizations and reference laboratories.

The reporting phase is a critical component of the LMT-Core assessment. This is the moment when assessed laboratories receive their comprehensive reports, which include a detailed analysis of their LMT results and expert recommendations from the assessors with a clear plan to guide improvements and next steps. Thus, in this regional analysis, we found that the *General laboratory profile* area of the 32 veterinary laboratories had a mean value of 68% (min: 33%, max: 90%). In general, the physical location of laboratories is highly satisfactory (71%; min: 22%, max: 100%). Most are located outside city centers, in closed compounds, and near major airports and highways. Most laboratories in the region have a good organizational structure with clear organizational charts (93%; min: 33%, max: 100%), well-defined terms of reference for their staff, and access to sufficient basic laboratory supplies (73%; min: 19%, max: 100%). A general gap observed across several laboratories in ASEAN is the lack of adequate laboratory budgets provided by their respective governments. Hence, there is a need for more advocacy for the authorities and governments to allocate funds to improve the functionality of their veterinary laboratories (20). The focus of laboratory capacity-building projects in ASEAN should aim at strengthening veterinary laboratory policy, laboratory leadership and management, and laboratory directors’ ability to develop operational and financial business plans for the laboratories under their supervision (21, 22). Improvement in the laboratory budget category requires skills in laboratory leadership and management. Indeed, higher managerial capacity is an asset for financial advocacy to stakeholders and competent authorities (23). Laboratories are also encouraged to invest time in writing grant applications for specific development or research programs to secure funding.

The mean value for the *infrastructure, equipment, and supplies* area is 60% (min: 23%, max: 83%), and the category that scored the lowest in this area is *infrastructure* (53%; min: 26%, max: 74%). This category is the sole category that did not display one laboratory scoring above 80% (very high in dark green) and only eight laboratories scored between 60 and 80%, suggesting that overall infrastructure quality and access to maintenance should be unanimously improved. Indeed, the other twenty-four needs investments in their overall infrastructure (such as renovations and refurbishments). Improvements in the infrastructure category relies on the regular access to building maintenance and infrastructure upgrades. Laboratories need sufficient space for a proper set-up and the implementation of the different departments, e.g., bacteriology, parasitology, serology, molecular, virology, post-mortem, histopathology, cleaning rooms with autoclaves and other equipment that require sufficient space. Laboratory design is thus essential to optimize workflow and minimize the risk of contamination (24). Laboratories must have a cleaning plan in place with written procedures and an appropriate ventilation system. Recently, the ISO/TC 336 for laboratory design was established to provide guidelines for the design and operation of functional, safe, energy-efficient, and sustainable laboratories (25). The laboratory infrastructure should have an emergency exit and adapted equipment to monitor any usual events, prevent and respond to accidents (26). This gap in infrastructure quality could be explained by the fact that (i) project funding usually covers for equipment and reagents but do not cover for laboratory renovation and (ii) governmental financial support to maintain an optimal laboratory structure is often missing (20, 27). For the *equipment* and the *reagents* categories, the mean values are higher than for the infrastructure, 59% (min: 13%, max: 96%) and 67% (min: 30%, max: 96%), respectively. Importantly, several laboratories scored very high results (>80%). One of the main recommendations when acquiring new equipment is for laboratories to aim, as much as possible, to buy equipment of higher quality from well-known manufacturers, which often include more extended warranties, a technical support service and assistance for maintenance. Unfortunately, most of the time, laboratories are constrained to buy the cheapest items (via a tender), and they end up using equipment of poor quality, which is often impossible to fix because of the costs of reparation and the lack of spare parts and customer service (9). As a matter of fact, procurement tender usually implies the purchase of the cheapest equipment available on the market, which has a low index of reparability and no access to customer service and affordable maintenance programs. This cheaper equipment does not last long, and as a result, some laboratories have accumulated many out-of-service or broken equipment in their facilities. Improvements in the *equipment* category can be mainly achieved by purchasing higher quality equipment and improving the access to frequent maintenance and calibration with certification of key equipment such as biosafety cabinets (BSCs), autoclaves, microscopes, and PCR machines. To this end, FAO has signed a long-term agreement with a well-known BSC manufacturers to improve access to maintenance of BSC and fume hood. Another observation made by LMT-Core assessors is that modern equipment is often unequally distributed amongst the different departments. Indeed, equipment will be preferentially provided to certain teams based on the importance and the prevalence of the diseases and also based on project-specific external funding. For instance, in some laboratories, virology departments are well-equipped due to Highly Pathogen Avian Influenza (HPAI) or Foot and Mouth Disease (FMD) programs, whilst parasitology departments use mostly outdated equipment. Bacteriology departments also receive more attention due to the emergence of awareness about anti-microbial resistance (AMR). However, they are still behind virology departments, where significant investments and training are regularly provided for zoonoses and emerging infectious diseases. Over the years, major international programs implemented in the region included complete rewiring of some laboratories with new generators and UPS, incinerators, renovation of the bench areas and supply of many different type of equipment including fridges, freezers, biosafety cabinets, microscopes, thermocyclers. Those programs also included some technical assistance. Consequently, the Infrastructure, equipment, and supplies increased steadily by 12%, with improved facilities (10%), new equipment (17%) and reagents (11%) installed by various projects and continued investments in surveillance programs.

Most laboratories in ASEAN have problems with their laboratory performance; the mean value for this regional analysis is 57%, which is the lowest area result. The main factor that impacted this result was access to samples (47%). Specific efforts should now be invested towards this specific gap as it remained unchanged over the years (Δ score = −3%). Laboratories are the best advocates for improved sample collection for testing. Addressing this gap is not simple, it means establishing a strong collaboration with other veterinary diagnostic laboratories and government agencies to share best practices about animal and emerging diseases to encourage (i) local communities (ii) veterinary staff and (iii) stakeholders to all engage in both passive and active surveillance programs. By developing clever strategies, countries can create a robust, efficient, and inclusive system for animal sample collection, ultimately improving disease detection and response (28–30). The staff skill and availability category scored 64% (min: 29%, max: 100%) in this regional analysis, and the trend over the years was stable (Δ score = 6%). In most laboratories, our observation is that senior staff are well-trained. However, there is a lack of adapted formation and training of the new generation staff, particularly when critical staff retires. As laboratory experts, we often find that it is easier to train new staff in laboratories with high-throughput activities so they can practice routine testing procedures repeatedly. Importantly, technical staff must be fully trained when working with potentially high-risk pathogens, both from the technical perspective and the BSS. Through the LMT-Core evaluation, we saw that there is a lack of a well-developed internal training program to ensure (i) that skills are not lost and (ii) harmonization of training amongst staff. Importantly, most laboratories do not have documented training programs, which is crucial for quality purposes; specific activities developed by FAO in the region are currently focusing on these training issues.

Regarding the Quality assurance and biosafety/biosecurity area, the mean value = 60% is in the high range. To our understanding, a few years ago, when the first LMT evaluations took place, many laboratories had not necessarily made QA and BSS a priority and received little support. Over the past decade, considerable efforts and external funding have been implemented to improve the quality assurance of the laboratories by implementing quality management system (QMS) to improve sample testing reliability and optimal results analysis (31–33). Similarly, biosafety and biosecurity procedures in ASEAN veterinary diagnostic laboratories have also been improved over the years by the implementation of specific programs (21, 34, 35) and indeed, the Δ score for this area of LMT-Core increased by 15%). For example, between 2012 and 2014, FAO developed a capacity-strengthening program focusing on quality assurance and biosafety, e.g., support programs implemented which aimed to obtain partial ISO17025 accreditation. One of the biggest hurdles for the laboratories is finding dedicated staff to hold key positions, such as quality managers and biosafety managers. The impacts of the various programs are clearly shown in this report since the Δ score for the QA category is 15% and BSS is 14%. Although the results here showed that only 2 laboratories were in the red scores (<40%) for QA and 3 laboratories for BS/BS. More attention and investments are still needed to the lower-score laboratories to increase their current level. A future challenge will be the maintenance of the current QA and biosafety/biosecurity levels in laboratories, mainly dependent on external resources to maintain their systems. Importantly, more capacities were also given to ensure better access to occupational health services for the laboratory staff, including the requirement for a specific vaccination scheme prior to a possible manipulation of potentially dangerous samples; the Δ score of this category was the highest with + 31% (Figure 10).

Laboratory collaborations and networking within the ASEAN region have increased over the last decade (Δ score for the area = 15%). The establishment of the ASEAN Laboratory Directors Forum (ALDF) in 2013 and the annual Laboratory Technical Advisory Group (Laboratory-TAG) meetings stimulated networking within the region (36). They increased networking and collaborations of ASEAN veterinary laboratories at inter-regional and global levels (Δ score for the category lab collaboration = 30%). In 2020, ASEAN finalized and endorsed the terms of reference for the ASEAN reference laboratories (ARLs). Currently, five laboratories are designated as ARL: NIAH in Thailand for brucellosis, FMD-RRL in Thailand for FMD, VRI in Malaysia for avian influenza, and RAHO-6 and NCVD in Viet Nam for swine diseases and rabies, respectively. These laboratories are steadily increasing their regional activities, such as organizing regional proficiency testing programs, testing and analyzing samples for confirmatory diagnosis, and providing training support to ASEAN member states (37). Some of these reference laboratories will improve their international laboratory networking over the coming years. Changing regulations for the importation of diagnostic samples and increased involvement in ALDF will also improve scores in this category.

The In-Country analysis revealed several situations; some laboratories were assessed only once, some countries had one laboratory assessed, and others had several labs assessed multiple times. Regardless of the situation, sole or multiple LMT-Core assessments with their associated results can always be exploited, and specific recommendations can be drawn. Most laboratories demonstrated encouraging trends of improvement in all LMT-Core areas. Results not openly displayed in this article (for data protection and privacy reasons) suggest that Central Veterinary Laboratories/Reference laboratories tend to score higher than sub-national, rural, local or satellite laboratories within a given national network, indicating that the repartition of the resources should be more spread out and importantly, that more efforts and attention should also be invested to all the laboratories including the smaller ones. For many years, the FAO has developed a strategy to implement specific capacity-building activities aimed at improving the core capabilities and capacities of veterinary laboratories in the ASEAN region (37). The FAO-LMT Core contributes to this strategy by setting a baseline evaluation at national and regional levels from which specific capacity-building activities can be implemented. Regular use of the FAO-LMT Core allowed the evolution measurement of the targeted activities, and hereby, the steady increase of most laboratories assessed are the results of the targeted efforts from FAO and many other local, national or international counterparts. These efforts must be continued to maintain the levels and further improve to higher standards.

## Data Availability

The original contributions presented in the study are included in the article/Supplementary material, further inquiries can be directed to the corresponding author.

## Author’s note

This consensus title represents the ASEAN Laboratory Directors of the diagnostic facilities involved in this study.
