# Supplementary material for: FAO laboratory mapping tool results analysis for veterinary laboratories from 2012 to 2020: highlights of the gaps, the strengths across Southeast Asia and implications for capacity building activities
Source: Front Vet Sci. 2026 Mar 4;12:1677993. doi: 10.3389/fvets.2025.1677993 (PMC12997447; doi:10.3389/fvets.2025.1677993)
Supplement: Supplementary file 1 [file Table_1.docx]

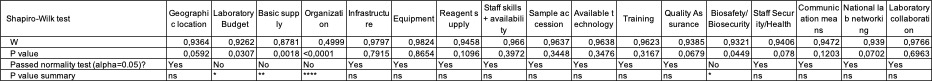


Supplementary table 1: Normal distribution of the 32 values according to the Shapiro-Wilk test. In the Shapiro-Wilk test, the W statistic (or W value) is a measure of how closely your sample data resembles a normal distribution. W ranges between 0 and 1 with W = 1: Perfect normality (data follows a normal distribution exactly) and W < 1: Deviations from normality (the smaller the W, the stronger the evidence against normality). For the interpretation - a high W (close to 1) suggests the data is normally distributed whilst a low W (far from 1) suggests the data is not normally distributed. Finally, the test also provides a p-value. If p < 0.05, you reject the null hypothesis (data is not normal).
