# Supplementary material for: FAO laboratory mapping tool results analysis for veterinary laboratories from 2012 to 2020: highlights of the gaps, the strengths across Southeast Asia and implications for capacity building activities
Source: Front Vet Sci. 2026 Mar 4;12:1677993. doi: 10.3389/fvets.2025.1677993 (PMC12997447; doi:10.3389/fvets.2025.1677993)
Supplement: Supplementary file 2 [file Table_2.docx]

Supplementary table 2. Scores of the LMT-Core (%) evaluation of the 32 laboratories involved in this study. The table shows the raw scores for each laboratory and the mean value per category, per area and for the whole LMT-Core. The colour coding system is as follow: 0- 20,0% = very weak (dark red); 20,1- 40,0% = weak (red); 40,1- 60,0% = average (orange); 60,1 – 80,0% = strong (light green); 80,1 - 100% = very strong (dark green).
