# Supplementary material for: FAO laboratory mapping tool results analysis for veterinary laboratories from 2012 to 2020: highlights of the gaps, the strengths across Southeast Asia and implications for capacity building activities
Source: Front Vet Sci. 2026 Mar 4;12:1677993. doi: 10.3389/fvets.2025.1677993 (PMC12997447; doi:10.3389/fvets.2025.1677993)
Supplement: Supplementary file 3 [file Table_3.docx]

Supplementary table 3. Delta scores (%) of the 18 laboratories for which 2 assessments were performed.
